# Preparation of SiO_2_-MnFe_2_O_4_ Composites via One-Pot Hydrothermal Synthesis Method and Microwave Absorption Investigation in S-Band

**DOI:** 10.3390/molecules24142605

**Published:** 2019-07-17

**Authors:** Pengfei Yin, Limin Zhang, Jian Wang, Xing Feng, Liang Zhao, Hanbing Rao, Yanying Wang, Jianwu Dai

**Affiliations:** 1 College of Science, Sichuan Agricultural University, Ya’an 625014, China; 2 Key Laboratory of Space Applied Physics and Chemistry (Ministry of Education), School of Science, Northwestern Polytechnical University, Xi’an 710072, China; 3 College of Mechanical and Electrical Engineering, Sichuan Agricultural University, Ya’an 625014, China

**Keywords:** manganese ferrite, silica, composites, microwave absorption performance, S-band

## Abstract

MnFe_2_O_4_ NPs are successfully decorated on the surface of SiO_2_ sheets to form the SiO_2_-MnFe_2_O_4_ composite via one-pot hydrothermal synthesis method. The phase identification, morphology, crystal structure, distribution of elements, and microwave absorbing properties in S-band (1.55~3.4 GHz) of the as-prepared composite were investigated by XRD, SEM, TEM, and Vector Network Analyzer (VNA) respectively. Compared with the pure MnFe_2_O_4_ NPs, the as-prepared SiO_2_-MnFe_2_O_4_ composite exhibits enhanced microwave absorption performance in this frequency band due to the strong eddy current loss, better impedance matching, excellent attenuation characteristic, and multiple Debye relaxation processes. The maximum reflection loss of −14.87 dB at 2.25 GHz with a broader −10 dB bandwidth over the frequency range of 1.67~2.9 GHz (1.23 GHz) can be obtained at the thickness of 4 mm. Most importantly, the preparation method used here is relatively simple, hence such composite can be served as a potential candidate for effective microwave absorption in S-band.

## 1. Introduction

In recent decades, with the rapid development of military and commercial radars and other electronic equipment, the electromagnetic pollution has become one of serious problem following the water, air and noise pollutions [1,2,3,4]. Currently, the preparation of microwave absorbers with high absorption strength, wide absorption bandwidth, low cost, simple and efficient is a key of the research in microwave absorption materials (MAMs). Much attention has been focused on the microwave absorbing materials of magnetic metals [5,6], dielectric materials [7,8,9], conductive macromolecules [10,11], ferrites [12,13] and so on. In which the ferrites have been widely used as the microwave absorber due to the special magnetic property, high Curie temperature, thermo-stable, anti-abrasion, and lower cost etc. [14]. As a kind of magnetic recording materials, the MnFe_2_O_4_ is a common ferrite with spinel structure and has been used in the field of microwave absorption for the past few years [15]. Such as M.P. Reddy et al. [16] synthesized the MnFe_2_O_4_ ferrite by a facile hydrothermal route and then consolidated into dense nanostructured compacts by the spark plasma sintering technique, the magnetic analysis indicated that the MnFe_2_O_4_ ferrite showed ferromagnetic behavior and can be applied in the microwave absorbing area.

However, to the best of our knowledge, the microwave absorption performance of unilateral MnFe_2_O_4_ is unsatisfactory due to the single magnetic loss mechanism. Hence, the MnFe_2_O_4_ ferrite can be composited with other materials to improve its electromagnetic wave absorbing properties. Y. Wang et al. [17] synthesized Ag/MnFe_2_O_4_/RGO and characterized the morphology, microstructure, electromagnetic properties of as-prepared composite, the results displayed that the maximum reflection loss reaches −38 dB at 6 GHz with the thickness of 3.5 mm. X.J. Zhang et al. [18] prepared the RGO/MnFe_2_O_4_/PVDF composite under the ultrasonic treatment, the maximum RL of the hybrid can achieve −29 dB at the frequency of 9.2 GHz with 5 wt% content, and the effective bandwidth of RL < −10 dB is in the range of 8~12.88 GHz. S.H. Hosseini et al. [19] synthesized polythiophene nanofibers coated MnFe_2_O_4_/Fe_3_O_4_ core-shell nanoparticles via co-precipitation and *in-situ* polymerization, the maximum microwave absorption of the product is −21 dB at 12 GHz. Y. Wang et al. [20] decorated the MnFe_2_O_4_ nanoparticles on the surface of reduced graphene oxide through a simple hydrothermal method, the maximum reflection loss of MnFe_2_O_4_/RGO composite is −32.8 dB at 8.2 GHz, and the absorption bandwidth with the RL below −10 dB is between 7.2~12 GHz. R.V. Lakshmi et al. [21] prepared the PMMA modified MnFe_2_O_4_-polyaniline nanocomposites with enhanced microwave absorption properties in the frequency range of X-band. H. Wang et al. [22] synthesized the MnFe_2_O_4_/wood composite by using a solvothermal method through a bottom-up pathway, the as-prepared hybrids exhibited an effective microwave absorption and the maximum reflection loss reached −12 dB at 15.52 GHz. S.H. Hosseini et al. [23] also prepared a PANI/MnFe_2_O_4_ nanocomposite with the core-shell structure, a maximum reflection loss of −15.3 dB was observed at 10.4 GHz. 

In conclusion, it is not difficult to find that the most existing research results of MnFe_2_O_4_-based composite are mainly focused in the range of the 2~18 GHz, and the microwave absorption performance of which would be much worse in the low frequency of S-band. However, the working frequency range of many radars has been extended to S-band and the radiant frequency of microwave generated from much electronic equipment used in our daily life is also seated in this range. Hence, the development of MnFe_2_O_4_-based composite with strong absorption ability in this band is intensively demanded [24,25].

In this work, the SiO_2_ sheets were introduced to composited with MnFe_2_O_4_ nanoparticles (denoted as MnFe_2_O_4_ NPs henceforth) via hydrothermal synthesis method. Therefore, the values of permeability and permittivity in composite can be modified to suitable range for enhancing the impedance matching properties of the absorber, which is very helpful to reinforce the microwave absorption of ferrite. The phase identification, morphology, crystal structure, distribution of elements and microwave absorbing properties in S-band of the as-prepared composites were investigated, the samples can obtain enhanced electromagnetic absorption performance in this frequency band compared with pure MnFe_2_O_4_ ferrite. It is believed that such composite can be served as a potential candidate of microwave absorber in the frequency range of S-band.

## 2. Results and Discussion

**Figure 1:**
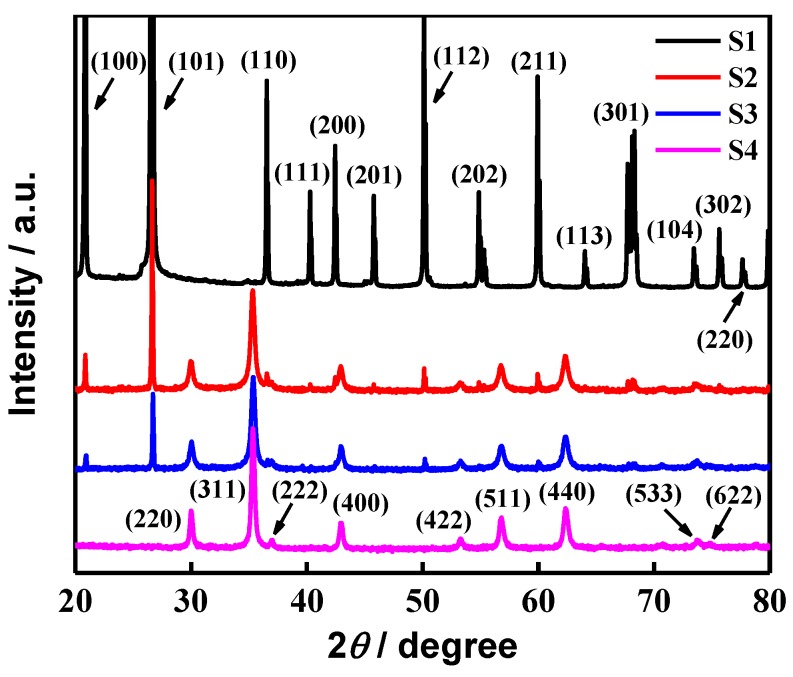
X-ray diffraction patterns of S1, S2, S3, and S4 samples.

Figure 1 displays the X-ray diffraction patterns of S1, S2, S3, and S4 samples. The characteristic diffraction peaks of S1 at 2*θ* = 20.86°, 26.64°, 36.54°, 40.30°, 42.45°, 45.79°, 50.14°, 54.87°, 59.96°, 64.03°, 68.32°, 73.47°, 75.66°and 77.67° are assigned to the (100), (101), (110), (111), (200), (201), (112), (202), (211), (113), (301), (104), (302) and (220) planes of SiO_2_ reported in the standard card (JCPDS card no.46-1045). Also, the diffraction peaks of S4 at 2*θ* = 29.71°, 34.98°, 36.65°, 42.53°, 52.74°, 56.20°, 61.66°, 72.92° and 73.93° are in good agreement with the (220), (311), (222), (400), (422), (511), (440), (533) and (622) planes of MnFe_2_O_4_ reported in the standard card (JCPDS card no. 10-0319), confirming the spinel structure of this ferrite. Moreover, it shows that the characteristic diffraction peaks in S2 and S3 samples corresponding to both SiO_2_ sheets and MnFe_2_O_4_ ferrite can be seen clearly, and no other diffraction peaks corresponding to impurities can be found as well, which indicates that the SiO_2_ sheets are composited well with the MnFe_2_O_4_ ferrite.

**Figure 2:**
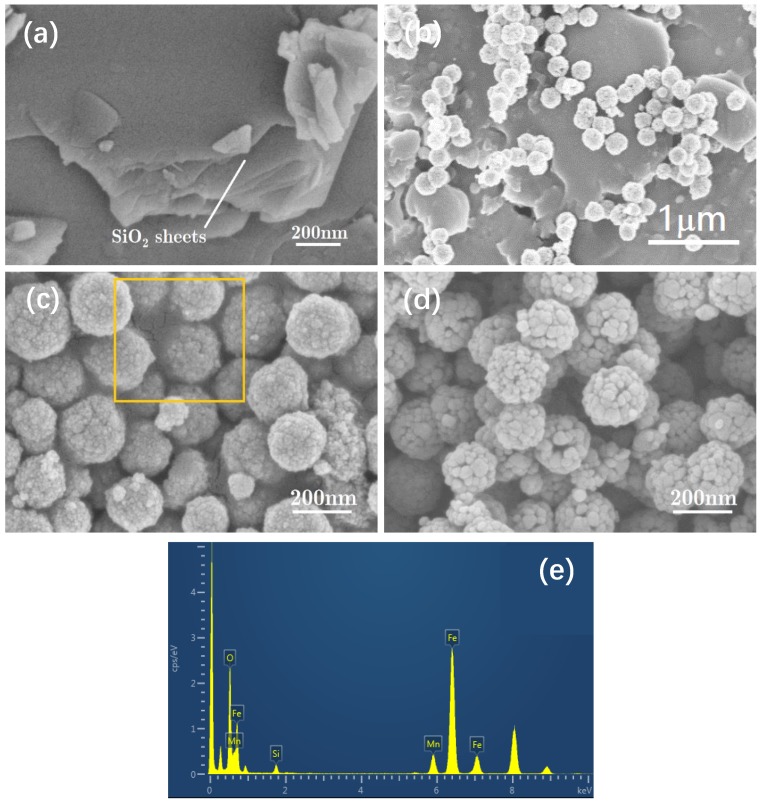
SEM images of sample S1 (**a**), sample S2 (**b**), sample S3 (**c**), sample S4 (**d**) and the Energy Dispersive Spectrometer (EDS) spectrum of sample S3 (**e**).

The microstructures of S1, S2, S3, and S4 are characterized by SEM and shown in Figure 2. It indicates that the raw silica presents a stacked lamellar structure in Figure 2a, while the fine MnFe_2_O_4_ NPs exhibits a rough ball-like structure with different particle sizes, and the average size of which is ~150 nm. As shown in Figure 2b, the ferrite NPs are adhered on the surface of SiO_2_ sheets and hard to cover the whole surface in general for the excessive silica content added. However, in the case of lower silica content, the ferrite NPs coated the surface of SiO_2_ sheets well, and even seems to be embedded into the surface as shown in the square region of Figure 2c. This tight bond between the two components is conducive to enhance the interfacial polarization effect of absorption materials, indicating that the S3 sample may have good microwave absorbing performance. Besides, it is interesting to note that the pure MnFe_2_O_4_ NPs in Figure 2d are consist of many smaller nanoparticles and stacked up together to form the loose structure. Moreover, the Energy Dispersive Spectrometer (EDS) of S3 sample was given in Figure 2e, suggesting the existence of Mn, Fe, O and Si elements in the composite, which can further confirm the possible associativity of SiO_2_ and MnFe_2_O_4_ NPs to a certain degree.

**Figure 3:**
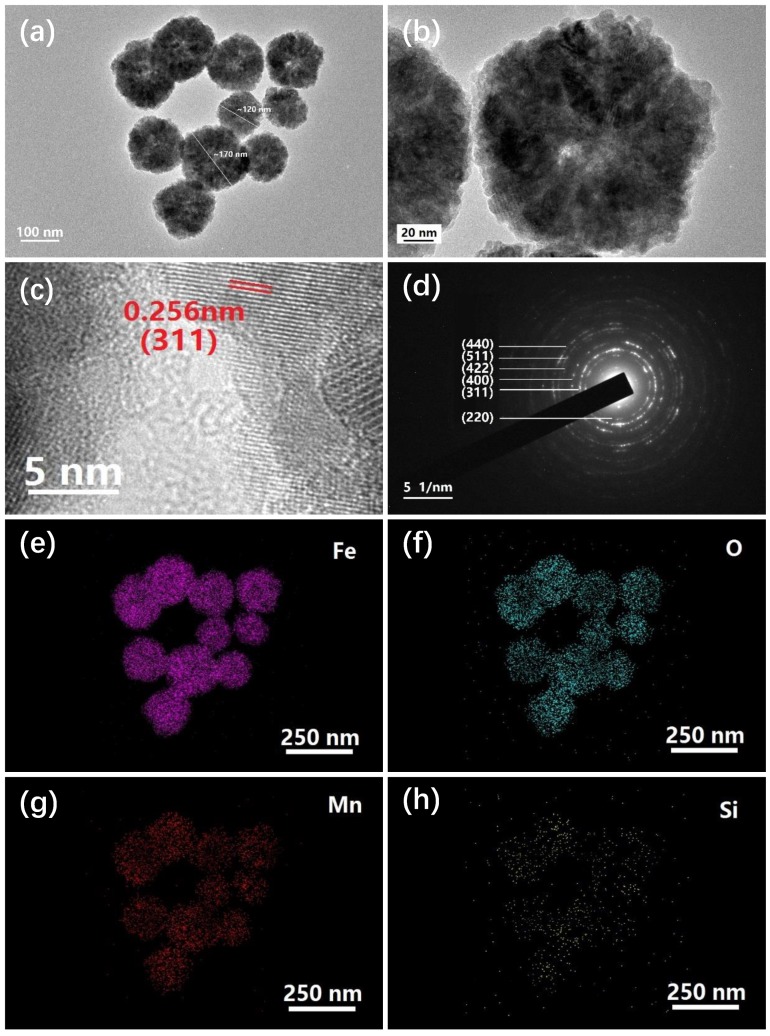
TEM images with different magnifications (**a**) and (**b**), HR-TEM image (**c**), SAED pattern (**d**) and corresponding elemental mapping images of S3 sample (**e**)~(**h**).

The transmission electron micrograph was used to further analyze the characteristics of NPs in S3 sample, as shown in Figure 3a, typical MnFe_2_O_4_ NPs show a diameter range of 120~170 nm and some of which appear to be hollow as shown in Figure 3b, each particle is made up of many smaller micro-crystals with different orientations. The HR-TEM image presented in Figure 3c exhibits clear lattice fringes with a spacing of 0.256 nm, which is corresponding to the interplanar spacing of (311) plane in NPs, indicating that the well crystalline nature of MnFe_2_O_4_ NPs. Figure 3d shows the selected area electron diffraction pattern of NPs, it describes some diffraction rings from inside to outside, which are assigned to the (220), (311), (400), (422), (511) and (440) planes of NPs respectively, further confirming the polycrystalline properties of NPs. Furthermore, the distribution of elements on NPs can be determined from the corresponding elemental mapping images from Figure 3e~h, the NPs are mainly consist of Mn, Fe and O elements, the small quantity of Si element is diffused from SiO_2_ sheets, and the distribution area of which is slightly larger than the other elements.

**Figure 4:**
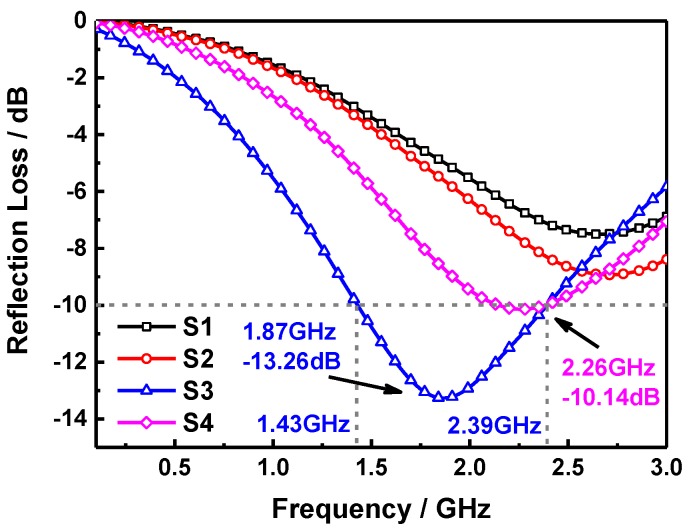
Frequency-dependent reflection loss (RL) curves of S1, S2, S3, and S4 at the thickness of 5 mm.

**Figure 5:**
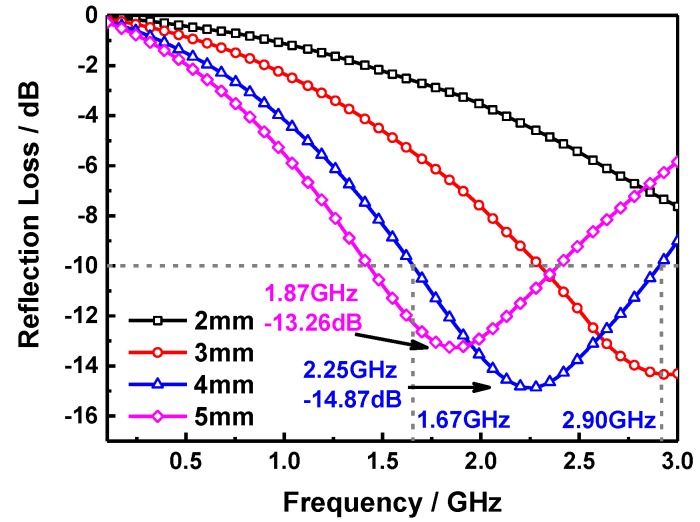
Frequency-dependent RL curves of S3 at different thicknesses.

In order to investigate the microwave absorption property of as-prepared samples, the microwave reflection loss (denoted as RL henceforth) is calculated according to the transmission-line theory, which can be defined with the following equations [26,27]:

(1) $$RL=20\log\left|\frac{Z_{in}-Z_{0}}{Z_{in}+Z_{0}}\right|$$

where *Z_in_* is the input impedance and can be expressed as:

(2) $$Z_{in}=Z_{0}\sqrt{\frac{\mu_{r}}{\varepsilon_{r}}}\tanh\left(j\frac{2\pi fd}{c}\sqrt{\mu_{r}\varepsilon_{r}}\right)$$

where, *ε_r_* is the complex permittivity, *μ_r_* the complex permeability, *c* the velocity of light in vacuum, *d* the thickness of samples, *f* the frequency and *Z*_0_ the impedance of air. Figure 4 shows the RL curves of S1, S2, S3, and S4 samples at a thickness of 5 mm, it can be seen that the microwave absorption performance of pure SiO_2_ sheets i.e., S1 sample is relatively limited and the maximum RL value is −7.50 dB at 2.67 GHz, which is due to the weak dielectric loss ability of SiO_2_. This situation will be improved as the SiO_2_ sheets are composited with the MnFe_2_O_4_ ferrite, the maximum RL value of S2 sample with the SiO_2_ mass content of 25 wt% reaches −8.93 dB at 2.70 GHz, for the single magnetic loss absorption of MnFe_2_O_4_ NPs is slightly stronger than the former, and the maximum RL of S4 is −10.14 dB at 2.26 GHz. It is interesting to note that the microwave absorption performance can be enhanced significantly in S3 sample i.e., the SiO_2_ mass content is 12.5 wt%. The maximum RL of which is −13.26 dB at 1.87 GHz with a −10 dB absorption bandwidth over the frequency range of 1.43~2.39 GHz (0.96 GHz). It indicates that the suitable composite ratio of SiO_2_ sheets and MnFe_2_O_4_ ferrite endows the sample a better impedance matching property and the excellent synergistic effect of dielectric loss and magnetic loss mechanisms. Furthermore, the interfacial polarization and associated relaxation between SiO_2_ sheets and MnFe_2_O_4_ can reinforce the dielectric loss as well [28]. These are all very helpful to improve the microwave absorption ability of absorber. As we all know that when the RL is below −10 dB, above 90% microwave energy will be dissipated during the transmission process in composites, thus the S3 sample possesses a good electromagnetic wave absorption performance in the S-band. Figure 5 shows the RL curves of the S3 sample at different thicknesses, it suggests that there is none obvious absorption peak can be observed in the measurement range as the thickness is 2 mm, and the maximum RL value is −7.63 dB at 3 GHz. However, the absorption peak appears gradually with the increase of thickness in sample, the absorption peak intensity of S3 achieves −14.33 dB at 2.94 GHz and the effective absorption bandwidth corresponding to the RL below −10 dB is focus in the range of 2.3~3 GHz (0.7 GHz) when the thickness of sample is 3 mm. Besides, it can be noted that the absorption peak intensity is enhanced only a little to −14.87 dB at 2.25 GHz as the thickness further added to 4 mm, however, the absorption bandwidth below −10 dB has been greatly increased, which is focus in the range of 1.67~2.9 GHz (1.23 GHz), this is superior to the microwave absorption property of sample with the thickness of 5 mm, and can cover the most of S-band. Moreover, it indicates that the RL peak moves to a lower frequency domain as the thickness of absorber increased, which is owing to the quarter-wavelength resonance effect [29]:

(3) $$f=\frac{nc}{4d\sqrt{\left|\mu_{r}\right|\left|\varepsilon_{r}\right|}}$$

Where, *ε_r_* and *μ_r_* are the complex permittivity and permeability of samples, *d* the thickness of absorber, *c* the velocity of light in free space. Thus, the corresponding frequency of the lowest RL peak shifts to a lower frequency when the thickness of the microwave absorbing material increased.

**Figure 6:**
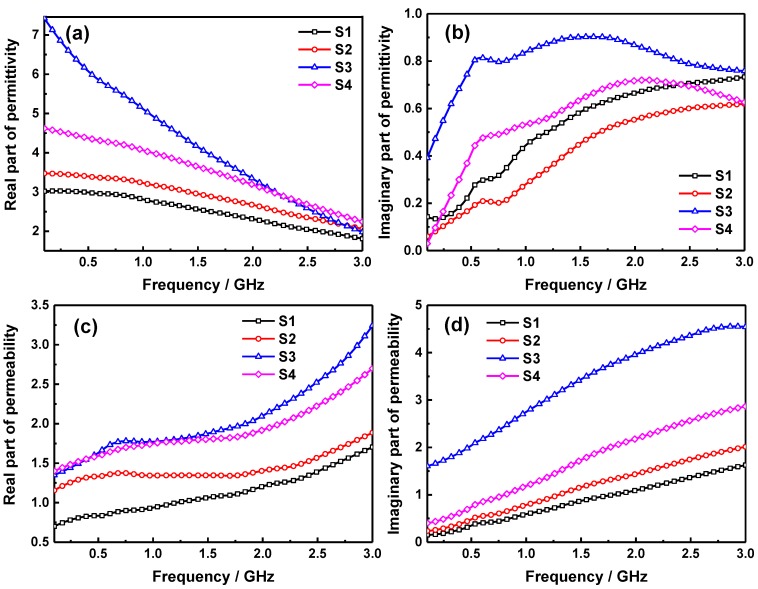
Frequency dependence of real part of permittivity (**a**), imaginary part of permittivity (**b**), real part of permeability (**c**) and imaginary part of permittivity (**d**).

In order to investigate the possible microwave absorption mechanisms, the frequency dependent complex permittivity and permeability of various samples were measured in the range and shown in Figure 6. As shown in Figure 6a, it can be seen that the real part of permittivity of S1, S2, S3, and S4 are all decreasing gradually with the increase of frequency in the measurement range. The *ε*′ of S1, S2 and S4 varies between 3.02~1.81, 3.47~2.06 and 4.62~2.24 respectively, which is heightened for the increase of MnFe_2_O_4_ content. However, the real part of permittivity of S3 is obviously higher than other three samples, especially in the low frequency domain, which varies from 7.43 to 1.98, indicating that the suitable doping of SiO_2_ in pure MnFe_2_O_4_ ferrite can be beneficial to heighten the *ε*′ of sample. While, in Figure 6b, the imaginary part of permittivity of S1 increases as the frequency increased, and the *ε*″ of S2 sample is lower than that of S1 during the entire frequency range in spite of *ε*″ of pure ferrite S4 is slightly larger than S1. It is interesting to note that the imaginary part of permittivity of S3 is obviously larger than those of other samples in the whole frequency range. This may be owing to the interfaces and defects generated from the recombination process of SiO_2_ and MnFe_2_O_4_ under the optimum ratio, thus the more polarization and related relaxation could be initiated around these interfaces and defects, which can further increase the imaginary part of permittivity in S3 sample. In general, the real part of permittivity represents the storage capacity of the microwave energy in absorbing materials, nevertheless the imaginary part of permittivity represents the energy loss ability [30]. Hence, the S3 sample may have an excellent microwave absorption performance in this frequency range. Moreover, the permittivity of sample in gigahertz range is closely related to its interfacial polarization and inherent dipole polarization, due to some resistance inside the material, the dipole reversal always can’t keep up with the speed of the electromagnetic field during the increase process of frequency, this will lead to the typical frequency dependent dielectric property of permittivity [31]. It can be seen from Figure 6c that the real part of permeability of S1, S2, S3, and S4 are all increasing continuously with the enhance of frequency, and it is heightened with the increase of ferrite content for the magnetic properties of the MnFe_2_O_4_ NPs. Moreover, the *μ*′ value of S3 is slightly greater than S4 in the high frequency part, this is ascribed to the fact that the dispersion and independence of MnFe_2_O_4_ NPs are greatly enhanced as the nanoparticles are embedded into the laminar SiO_2_ sheets, which is beneficial to exhibit the quantum scale effect of fine particles to enlarge the *μ*′ of S3. Furthermore, the change tendency of imaginary part of permeability in Figure 6d is mainly the same as that of the real part of permeability, however, the *μ*″ of S3 is much larger than pure MnFe_2_O_4_ ferrite, indicating that a higher magnetic loss property of the sample. As we all know that the imaginary part of permeability presents the magnetic loss ability of sample in general [32], and the natural resonance of magnetic loss can be expressed by the following equation [33]:

(4) $$2\pi f_{r}=\gamma H_{a}$$

(5) $$H_{a}=\frac{4\left|K_{1}\right|}{3\mu_{0}M_{s}}$$

Here, *H_a_* is the anisotropy energy, |*K*_1_| the anisotropic coefficient, *μ*_0_ the permeability in a vacuum, *M_s_* the saturation magnetization and *γ* the gyromagnetic ratio. On one hand, the anisotropy energy of nano-size materials would be remarkably increased due to the surface anisotropic field by the small size effect. On the other hand, the suitable compound proportion in S3 sample is also helpful to enhance its anisotropy energy. These are all beneficial to improve the magnetic loss of S3 sample.

**Figure 7:**
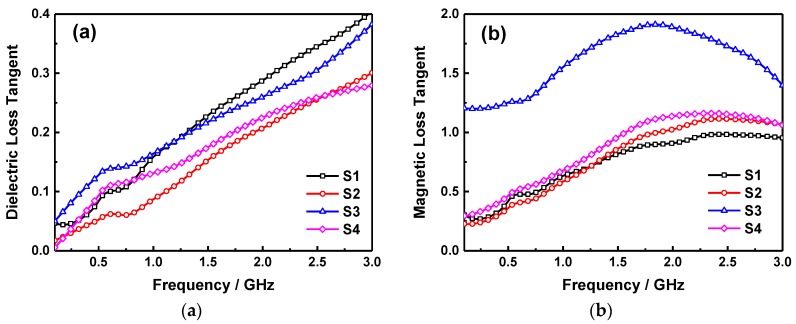
Frequency dependence of dielectric loss tangent (**a**) and magnetic loss tangent (**b**).

The dielectric and magnetic loss tangent of S1, S2, S3, and S4 samples can be calculated according to the permittivity and permeability measured above, which are shown in Figure 7. In Figure 7a, it indicates that the dielectric loss of the samples are all increasing as the frequency increased, which is relatively higher in the S1 than that of S4 for its dielectric property of raw SiO_2_ sheets, particularly in the high frequency domain. Although the dielectric loss in sample will recede as the two components composited together, however, the value of S3 get a slight boost in the low frequency part, it varies from 0.05 to 0.38 during the whole testing frequency range. As shown in Figure 7b, the magnetic loss of S1, S2 and S4 are enhanced with the increase of ferrite content, and mainly between 0.27~0.98, 0.23~1.11 and 0.29~1.16 respectively, moreover, there is an obvious peak value can be observed in the measurement range. While, the *μ*″ of S3 is significantly higher than other samples with a peak value of 1.91, indicating that the better magnetic loss property for microwave absorption of S3 sample, this is due to the reason mentioned above i.e., embedded structure of NPs in the sheets and enhanced anisotropic energy to improve the natural resonance of magnetic loss. In addition, it can be found that although the dielectric and magnetic loss are both contribute to the microwave absorption of samples, the effect of magnetic loss is more obvious for the relatively higher values.

Usually, the magnetic loss of ferrite mainly originates from hysteresis loss, eddy current loss, natural resonance loss and domain wall resonance loss. However, the domain wall resonance loss mainly occurs in the frequency range of megahertz, and the hysteresis loss is too weak to be neglected within a feeble field [34]. Hence, the natural resonance loss and eddy current loss are the most probable reasons for the electromagnetic absorption in gigahertz range, in which the eddy current loss can be expressed as follows [35]:

(6) $$\mu^{\prime \prime }\approx 2\pi \mu_{0}{\left(\mu^{\prime }\right)}^{2}\sigma d^{2}f/3$$

Here, *σ* is the electrical conductivity, *μ*_0_ the permeability in a vacuum. Hence, *C*_0_ can be expressed as follows [36]:

(7) $$C_{0}=\mu^{\prime \prime }{\left(\mu^{\prime }\right)}^{-2}f^{-1}\approx 2\pi \mu_{0}\sigma d^{2}/3$$

**Figure 8:**
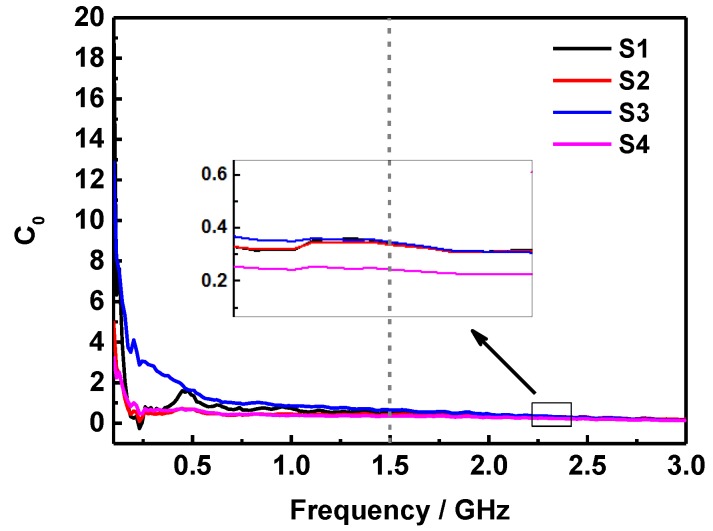
Frequency dependency of the eddy current loss curves of S1, S2, S3, and S4 at the thickness of 4 mm.

**Figure 9:**
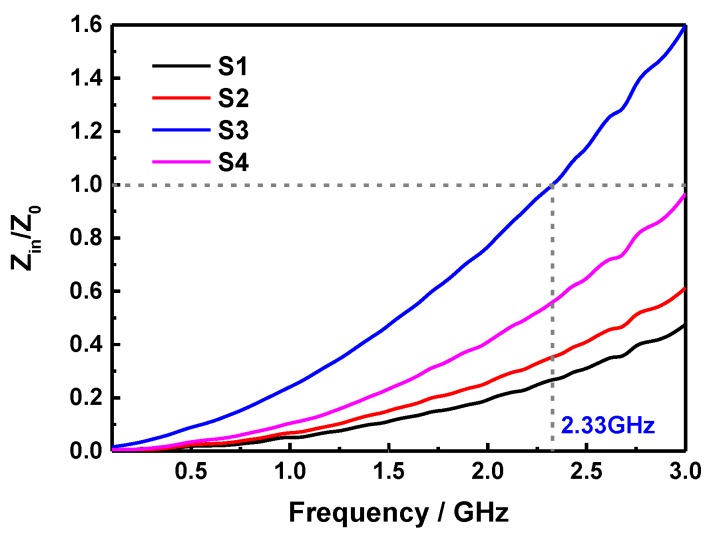
Frequency dependent Z_in_/Z_0_ values of S1, S2, S3, and S4 at the thickness of 4 mm.

If the reflection loss comes from the eddy current loss, the values of *C*_0_ will keep the constant when the frequency varies. Figure 8 shows the frequency-dependent *C*_0_ curves of S1, S2, S3, and S4 at the thickness of 4 mm. It can be noted that the value of *C*_0_ is almost constant in the frequency range of 1.5~3 GHz, confirming that the samples possess an obvious eddy current loss for the microwave energy absorption in this range. While, the magnetic loss in 0.1~1.5 GHz is mainly caused by the natural resonance, and the proper content of SiO_2_ sheets in S3 is conducive to enhance this effect. Moreover, the *Z_in_*/*Z*_0_ values of S1, S2, S3, and S4 with a thickness of 4 mm were calculated and shown in Figure 9. In principle, the perfect impedance matching demands that the value of *Z_in_*/*Z*_0_ is equal to 1, thus there is none reflection of microwave at the front surface of absorber can be realized [37,38]. As shown in Figure 9, it can be observed that the *Z_in_*/*Z*_0_ value of S3 sample with a layer of 4 mm is closer to the free space at about 2.33 GHz, while that of other samples are far lower than 1 in the range of 0.1~3 GHz, indicating that the excellent microwave absorption performance of S3 in the above frequency range, and more microwaves will transmit into the absorber to be dissipated.

The attenuation constant *α* is another important parameter related to the RL of samples, which determines the attenuation properties of microwave absorbing materials and can be determined according to the following equation [39,40]:

(8) $$\alpha =\frac{\sqrt{2}\pi f}{c}\times \sqrt{(\mu^{\prime \prime }\varepsilon^{\prime \prime }-\mu^{\prime }\varepsilon^{\prime })+\sqrt{{\left(\mu^{\prime \prime }\varepsilon^{\prime \prime }-\mu^{\prime }\varepsilon^{\prime }\right)}^{2}+{\left(\mu^{\prime }\varepsilon^{\prime \prime }+\mu^{\prime \prime }\varepsilon^{\prime }\right)}^{2}}}$$

**Figure 10:**
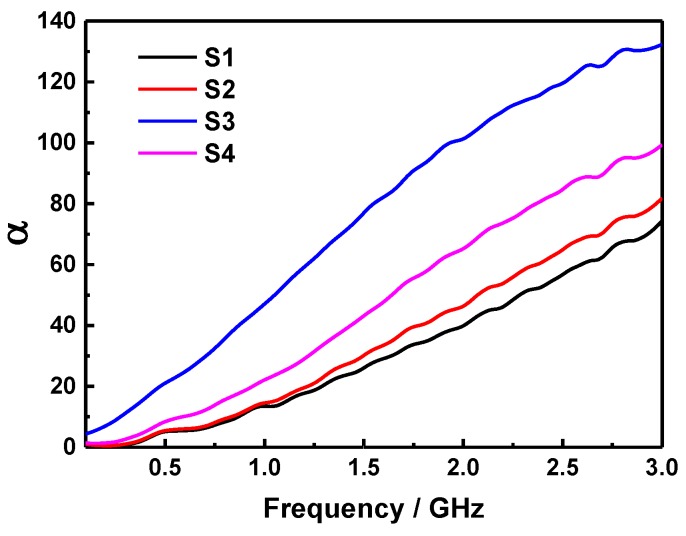
Attenuation constants of S1, S2, S3, and S4 at the thickness of 4 mm.

**Figure 11:**
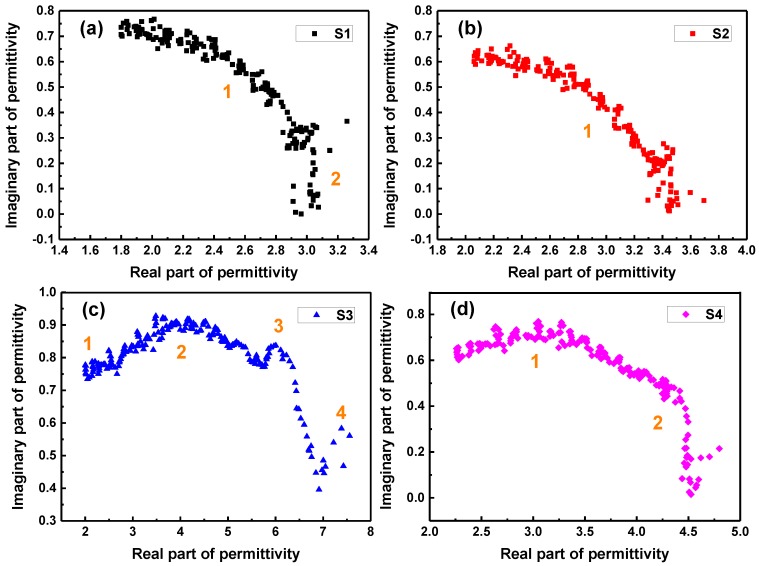
Plots of $\varepsilon^{\prime }-\varepsilon^{\prime \prime }$ for S1, S2, S3, and S4 at the thickness of 4 mm.

Figure 10 displays the attenuation constants of S1, S2, S3, and S4 at the thickness of 4 mm. It shows that the *α* is enhanced as the frequency increased in each sample, and that of S1 sample is the lowest among all the samples for its relatively lower imaginary part of permeability. In addition, the *α* of samples are strengthened with the increase of ferrite mass content, however, the S3 sample have a greater *α* than other samples in the whole frequency range owing to its higher values of *ε*″ and *μ*″, indicating that the outstanding attenuation characteristic is highly conducive to the electromagnetic wave absorption performance of the sample. Furthermore, the Debye dipolar relaxation is also an important factor for the dielectric loss of microwave absorber, according to the Debye theory the relationship between *ε*′ and *ε*″ can be exhibited as follows [41,42]:

(9) $${\left(\varepsilon^{\prime }-\frac{\varepsilon_{s}+\varepsilon_{\infty }}{2}\right)}^{2}+{\left(\varepsilon^{\prime \prime }\right)}^{2}={\left(\frac{\varepsilon_{s}-\varepsilon_{\infty }}{2}\right)}^{2}$$

where, *ε_∞_* is the dielectric constant at infinite frequency, and *ε_s_* the static dielectric constant. Thus, the curve of *ε*′ − *ε*″ is denoted as the Cole-Cole semicircle, which is corresponded to the Debye relaxation process. Figure 11 shows the *ε*′ − *ε*″ curves of S1, S2, S3, and S4 at the thickness of 4 mm. The results suggest that there are two Cole-Cole semicircles can be observed in S1 and S4 samples, confirming that two Debye relaxation processes have occurred during the microwave absorption process in the samples. Clearly, only one Debye relaxation process can be seen in the S2 sample for one obvious Cole-Cole semicircle. However, the S3 sample has much more different-sized semicircles than those of other samples, indicating that multiple Debye relaxation processes have been produced in the absorption of electromagnetic wave energy, which is one of significant reasons that the microwave absorption of S3 sample is greatly enhanced in the S-band.

Overall, the comparison of microwave absorbing properties between as-prepared SiO_2_-MnFe_2_O_4_ composite and other materials available in the literature have been listed in Table 1 [43,44,45,46,47,48,49,50]. It can be noted that the ferrites are often composited with other materials to enhance their impedance matching and electromagnetic absorption. Moreover, the comparison results suggest that most of the composites based on ferrites have excellent microwave absorption performance in GHz range, however, the absorbing frequency range of SiO_2_-MnFe_2_O_4_ composite prepared in this work is obviously the lowest compared with those obtained in other literatures.

## 3. Experimental

### 3.1. Materials

The manganese chloride tetrahydrate (MnCl_2_·4H_2_O) and ethylene glycol were purchased from Sinopharm Chemical Reagent Co. Ltd., China. The ferric chloride hexahydrate (FeCl_3_·6H_2_O), sodium acetate trihydrate (NaAc) and polyethylene glycol 6000 were purchased from Chengdu Kelong Chemical Reagent Co. Ltd., China. The SiO_2_ sheets were purchased from Beiyan Celiang Co. Ltd., China. All the chemicals were of analytic purity grade and were used as received without any further purification.

### 3.2. Preparation of MnFe_2_O_4_ NPs and SiO_2_-MnFe_2_O_4_ Composites

The SiO_2_-MnFe_2_O_4_ composites were prepared via typical one-pot hydrothermal synthesis method [51]. In the process, 6.75 g ferric chloride hexahydrate (25 mmol) and 2.475 g manganese chloride tetrahydrate (12.5 mmol) were added into 200 mL ethylene glycol with continuous stirring to form a clear solution. Then, 18 g sodium acetate trihydrate (132.35 mmol) and 5 g polyethylene glycol 6000 (~0.83 mmol) were consecutively added into the preceding solution with sustained stirring and ultrasonic dispersion for 30 min. Subsequently, the different masses of SiO_2_ sheets were put into the above solution and keep stirring for 10 min. Finally, the uniform solution was sealed into a 300 mL hydrothermal reactor and held the temperature at 180 °C for 12 h. After the reaction, the products were SiO_2_-MnFe_2_O_4_ composites and could be extracted out from the reaction liquid with a magnet. Then, the separated black precipitation was washed with ethanol to remove the impurities and dried in a drying oven at 50 °C for 6 h. In addition, the preparation of pure MnFe_2_O_4_ NPs was through the same process without the addition of SiO_2_ sheets. For the convenience of description, the four samples of pure SiO_2_ sheets, SiO_2_-MnFe_2_O_4_ composites with the SiO_2_ sheets mass content of 25 wt% and 12.5 wt%, and Pure MnFe_2_O_4_ NPs were marked as S1, S2, S3, and S4 respectively in the following text. By the way, take the S2 sample as an example, the quality of product, in theory, is 3.85 g. However, the actual mass of product is 3.10 g after drying sufficiently, thus the yield of this synthesis procedure is approximately to 80.52%.

### 3.3. Characterization

The crystal structures of S1, S2, S3, and S4 were analyzed by an X-ray powder diffraction system (German Bruker D8 with Cu-*K*_α_ radiation, *λ* = 0.154 nm). The Scanning Electron Microscope (SEM, SU8100, Hitachi, Japan) and Transmission Electron Microscope (TEM, Tecnai F30 G2, FEI, Morristown, NJ, USA) were employed to characterize the micromorphology and elements distribution of the samples. The samples used for electromagnetic wave absorption measurement were prepared by mixing the products with paraffin in a mass percentage of 50 wt%, for the composites would be very hard to shape as the powders were overloaded. Then, the produced samples were pressed into a toroidal shape (Φ_in_ = 3.04 mm and Φ_out_ = 7.00 mm) with different thicknesses and the S-parameters of samples were measured by a TIANDA TD3618C Vector Network Analyzer with coaxial transmission and reflection method. Finally, the relative complex permittivity and permeability of samples can be calculated according to the theory of Nicolson and Ross [52].

## 4. Conclusions

In summary, the SiO_2_-MnFe_2_O_4_ composite with significant microwave absorption performance in S-band was prepared via one-pot hydrothermal synthesis method, the microwave absorbing property of the as-prepared sample was also investigated. The dielectric and magnetic loss are both contribute to the microwave absorption of samples, while the effect of magnetic loss is more obvious. The preferable microwave absorbing ability of sample is attributed to the strong eddy current loss, better impedance matching, excellent attenuation characteristic and multiple Debye relaxation processes. Thus, the maximum reflection loss of −14.87 dB at 2.25 GHz with a broader −10 dB bandwidth over the frequency range of 1.67~2.9 GHz (1.23 GHz) can be obtained at the sample thickness of 4 mm. Most importantly, the preparation method reported here is relatively simple, make it quite a suitable candidate for the microwave absorption in S-band.

## Figures and Tables

**Table 1:** Comparison of microwave absorbing properties between SiO_2_-MnFe_2_O_4_ composite and other materials available in the literature.

Materials	Thickness	Minimum RL	Position	RL < −10 dB	Refs.
SiO_2_-MnFe_2_O_4_	4 mm	−14.87 dB	2.25 GHz	1.67~2.9 GHz	This work
ZnO/Fe_3_O_4_/GO	2 mm	−7.2 dB	/	6.4~8 GHz	[43]
PANI/Fe_3_O_4_/MWCNT	4 mm	−16 dB	/	8~15 GHz	[44]
Zn-doped CoFe_2_O_4_ cubes@CNT	2 mm	−9.98 dB	9.33 GHz	/	[45]
MWCNTs/Fe_3_O_4_	2 mm	−18.22 dB	/	10.9~12.4 GHz	[46]
CoFe_2_O_4_/NiFe_2_O_4_	4.5 mm	−20.1 dB	9.7 GHz	7.8~16.2 GHz	[47]
CoFe_2_O_4_/LPA-SWCNT	2 mm	−30.7 dB	12.9 GHz	10.1~17.3 GHz	[48]
Carbon-coated CoFe-CoFe_2_O_4_	3.93	−60 dB	5.5 GHz	4.1~7.8 GHz	[49]
CoFe_2_O_4_ fiber	5 mm	−36.5 dB	6.0 GHz	/	[50]
